# β-Glucan Combined With PD-1/PD-L1 Checkpoint Blockade for Immunotherapy in Patients With Advanced Cancer

**DOI:** 10.3389/fphar.2022.887457

**Published:** 2022-04-25

**Authors:** Mengjie Wang, Yu Bai, Jiaxin Pei, Dongqing Li, Xiaolin Pu, Wenyu Zhu, Lei Xia, Chunjian Qi, Hua Jiang, Yongling Ning

**Affiliations:** ^1^ Department of Oncology, The Affiliated Hospital of Nanjing Medical University, Changzhou No. 2 People’s Hospital, Changzhou, China; ^2^ Medical Research Center, The Affiliated Hospital of Nanjing Medical University, Changzhou No. 2 People’s Hospital, Changzhou, China; ^3^ Department of Oncology, Graduate School of Dalian Medical University, Dalian, China

**Keywords:** β-Glucan, PD-1, PD-L1, immune checkpoint blockade therapy, resistance

## Abstract

Programmed death-1 (PD-1)/PD-ligand 1 (PD-L1) checkpoint blocking antibodies have been shown to be a powerful immune checkpoint blockade (ICB) therapy for patients with cancer. However, patients quickly develop resistance to immunotherapy. β-glucan, an immune adjuvant, has been found to stimulate innate and adaptive immune responses. In this study, we assessed the use of whole glucan particle (WGP) β-glucan in combination with PD-1/PD-L1–blocking antibodies to slow down the resistance to immunotherapy. Results from a tumor mouse model demonstrated that administration of WGP β-glucan plus PD-1/PD-L1–blocking antibodies led to increased recruitment of immune-associated cells, improved regulation of the balance between T-cell activation and immune tolerance, and delayed tumor progression. This combination therapy was also found to improve progression-free survival in patients with advanced cancer who had previously discontinued anti-PD-1/PD-L1 because of disease progression. These findings suggest that β-glucan could be used as an immune adjuvant to reverse anti-PD-1/PD-L1 resistance by regulating the immune system.

## Introduction

Immune checkpoint blockade (ICB) therapy is an exciting new approach to cancer immunotherapy that has been increasingly studied in recent years. Research has shown that ICB can remit tumor cell–induced suppression in the tumor microenvironment (TME), taking advantage of immune cell infiltration in the tumor to reactivate an effective antitumor immune response (Kuzume et al., 2020; Petitprez et al., 2020).

Programmed death-1 (PD-1) (CD279), an inhibitory receptor that is expressed in activated T cells and B cells, suppressed innate and adaptive immune responses (Grabie et al., 2007). In particular, PD-1 is highly expressed on tumor-specific T cells. Research has shown that the inhibition of PD-1 promotes an effective immune response to suppress cancer aggression (Meng et al., 2006; Maier et al., 2007). PD-1 has two ligands: PD-L1 (B7-H1, CD274) and PD-L2 (B7-DC, CD273). PD-L1 in particular is seen in a variety of cell types, including dendritic cells (DCs), macrophages, and T and B cells (Muthumani et al., 2011). In addition, PD-L1 is expressed by tumor cells to escape antitumor responses (Han et al., 2020). Interactions between PD-1 and PD-L1 were initially reported to cause inhibitory signals mainly affecting T-cell responses (Paterson et al., 2011). More recent researches suggest that the PD-1/PD-L1 pathway plays a key role in stimulating T-cell activation and modulating tolerance in antitumor immune responses (Alsaab et al., 2017). In addition, the pathway is an essential mediator of tumor-infiltrating lymphocytes (TILs) in the TME; this mediation includes addressing T-cell exhaustion by controlling the host defenses aimed at T-cell unresponsiveness in the suppressive TME.

The introduction of drugs targeting the PD-1/PD-L1 pathway, including ICB therapy, has revolutionized the treatment of multiple types of cancer, including melanoma and lung cancer (Wang et al., 2020). Unfortunately, research has shown that patients quickly develop resistance to immunotherapy (Dammeijer et al., 2020), which is a significant complication for patients with advanced cancer. As PD-1/PD-L1–blocking antibodies apply to TILs in the TME, this therapy can reactivate the exhausted T cells and thereby recover pre-existing antitumor immune responses (Naidoo et al., 2015). Based on this fact, several hypotheses have been proposed to explain the lack of ICB efficacy in patients, such as lack of TILs in the TME. The transformation from a “cold tumor”to a“hot tumor” is a highly consistent consensus.

β-glucan, a macromolecular polysaccharide, is a major fungal pathogen-associated molecular pattern (PAMP) that stimulates innate and adaptive immune responses (Goodridge et al., 2009; Lee et al., 2016). The function of β-glucan has been demonstrated through the C-type lectin receptor dectin-1 or complement receptor 3 (CR3) (Tian et al., 2013). Dectin-1 is a DC-specific molecule that is mainly expressed on macrophages, monocytes, and neutrophils (Daley et al., 2017); CR3 is a member of the β2 integrin family and is highly expressed on neutrophils, monocytes, and NK cells (Ehlers, 2000; Doz-Deblauwe et al., 2019). As a natural immune adjuvant, β-glucan can be found in various sources with different structures and biological effects (Maity et al., 2015). Whole glucan particle (WGP) β-glucan, which is derived from yeast, has a long β-1,3-D-glucan backbone and β-1,6-glucan link side chains and has strong antitumor and anti-infection effects (Ding et al., 2015). Previous research has shown that oral WGP β-glucan purified from the cell walls of *Saccharomyces cerevisiae* can induce DC maturation, activate T cells, and stimulate cytokine secretion, leading to a delay in tumor progression (Qi et al., 2011; Ning et al., 2016; Ding et al., 2019).

To address the disadvantages of immunotherapy discussed above, we sought to investigate the use of WGP β-glucan plus PD-1/PD-L1–blocking antibodies as antitumor therapy. We hypothesized that WGP β-glucan would enhance the concentration of TILs in the TME and that PD-1/PD-L1–blocking antibodies would slow the development of tolerance to immunotherapy. To this end, we presented an experimental model to determine the therapeutic efficacy of oral WGP β-glucan combined with PD-1/PD-L1–blocking antibodies in lung cancer mouse. The efficacy and safety of this combination therapy was evaluated in patients with advanced cancer who had developed a tolerance to immunotherapy. The purpose of the study was to determine whether WGP β-glucan combined with PD-1/PD-L1–blocking antibodies could be used to augment and reinvigorate ICB therapy in patients with advanced cancer.

## Materials and Methods

### Whole Glucan Particle β-Glucan Synthesis

WGP β-glucan was purified from the cell walls of *S cerevisiae*. Through a series of alkaline and acid extractions, cytoplasm and other cell wall polysaccharides such as mannose were removed. An intact hollow yeast cell wall remained, composed primarily of long β-1,3 glucose polymers, with 3% to 6% of the backbone glucose units possessing a b (1,6) branch (b-(1,3/1,6)-D-glucan).

### Mice and Tumor Cell Lines

Female C57BL/6 mice aged 6–8 weeks were purchased from Changzhou Cavens Laboratory Animal Co., Ltd. All mice were maintained under specific pathogen-free conditions in individually ventilated cages. All mouse experiments were performed in compliance with all relevant laws and institutional guidelines and were approved by the Institutional Animal Care and Use Committee of Nanjing Medical University.

A Lewis lung carcinoma (LLC) cell line derived from C57BL/6 mice was provided by the American Type Culture Collection. The LLC cells were cultured in Dulbecco’s Modified Eagle’s Medium (DMEM) supplemented with 10% fetal bovine serum (Thermo Fisher Scientific) in a humidified atmosphere and at 5% CO_2_ in air. Culture flasks were used to reach the appropriate tumor cell concentrations for injection.

### *In Vivo* Experiments in Mouse Tumor Models

LLC cells (3 × 10^5^) suspended in 100 μL phosphate buffered saline (PBS; Thermo Fisher Scientific) were subcutaneously injected into the flanks of C57BL/6 mice. When the tumor masses were palpable (at approximately 6–7 days after injection), treatment was initiated. Mice were randomly assigned to 1 of 4 experimental groups: control (PBS), WGP, anti-PD1, or WGP + anti-PD1. The tumor masses were measured using a caliper every other day, and tumor volumes were calculated as follows:

V=12×the vertical length×the horizontal length2

Mice with established subcutaneous LLC tumors were euthanized when the tumor length reached 15 mm (at approximately 20 days after injection).

### Whole Glucan Particle and Anti-PD-1 Treatments in Mice

Mice in the WGP and WGP + anti-PD1 groups received 1 mg WGP in 100 μL PBS daily *via* oral administration. Mice in the anti-PD1 and WGP + anti-PD1 groups received 20 μg anti-PD-1 antibody (clone 29F.1A12, Biolegend) in 100 μL PBS once every 3 days *via* intravenous injection into the tail. Mice in the control group received 100 μL PBS.

### Surface and Intracellular Staining of Mice Tumor Samples

Surface staining and intracellular staining were performed as described previously. Briefly, tumor tissues were weighed and minced into small pieces. Then digested for 30 min at 37°C with rotation with a triple enzyme mixture containing collagenase type IV, hyaluronidase, and deoxyribonuclease (Sigma-Aldrich). Filtering was performed to remove insoluble fiber from the cells, and red blood cells were lysed with red blood cell lysis buffer (Beyotime Biotechnology). The cells were then washed twice with ice cold PBS.

### Flow Cytometry

For surface marker staining (Ning et al., 2016), single cell suspensions from mice tumors were stained with fluorescein-conjugated mouse-specific antibodies against CD11c, CD11b, F4/80, Gr-1, PD-1, PD-L1, CD45, CD3, CD4 or CD8 (Biolegend) at 4°C for 30 min in the dark. After the cells were washed twice with PBS, they were either directly measured or, for intracellular staining, fixated and permeabilized with Foxp3 (Biolegend) to stain nuclear factors. Intranuclear antibodies were then incubated at room temperature for 30 min in the dark. This was followed by washing and flow cytometry, which was performed on a FACS Canton II (BD Biosciences). Results were analyzed using FlowJo version 10 (Tree Star).

Immune cell subsets were characterized using the following sets of markers: DCs (CD11c^+^/CD8^+^), macrophages (CD11b^+^/F4-80^+^), myeloid-derived suppressor cells (MDSCs: CD11b^+^/Gr-1^+^), CD8^+^ T cells (CD45^+^/CD3^+^/CD4^-^/CD8^+^), CD4^+^ T cells (CD45^+^/CD3^+^/CD8^-^/CD4^+^), and Tregs (CD45^+^/CD3^+^/CD4^+^/Foxp3^+^).

### qRT-PCR

Tumor samples were treated with TRIzol reagent (Invitrogen, Thermo Fisher Scientific), and total RNAs were isolated and reverse transcribed with TaqMan Reverse Transcription Reagents (Applied Biosystems). The indicated cytokine mRNA levels were quantified with quantitative RT-PCR amplification using the BioRad MyiQ single-color RT-PCR detection system. Briefly, complementary DNA was amplified in a 20-μL reaction mixture containing 10 μL of SYBR Green PCR supermix (Invitrogen, Thermo Fisher Scientific), 100 ng of complementary DNA template, and selected primers (200 nM) using the recommended cycling conditions. Data were acquired on an ABI ViiA 7 real-time PCR system. The primer sequences, designed with Primer Express Software Version 2.0 (Applied Biosystems), are summarized in the Supplementary Table S1.

### Patient Cohort

This open label, one-arm pilot clinical study was registered with the Chinese Clinical Trial Registry (ChiCTR2100043913). Patients treated at Changzhou No. 2 People's Hospital affiliated to Nanjing Medical University were eligible for this study if they were aged 18 to 75 years; had a histopathological and/or cytological diagnosis of a distant metastases and advanced tumor; failed at least one-line regimens containing PD-1/PD-L1 inhibitor had ≥1 measurable lesion based on Response Evaluation Criteria in Solid Tumors (RECIST) 1.1 criteria; the expected survival ≥3 months; had no significant liver or renal impairment or severe cardiorespiratory disease; and had Eastern Cooperative Oncology Group (ECOG) performance status of 1 or 2. Patients were excluded from the study if they were enrolled in any clinical trials assessing other drugs within 4 weeks and if they had any factors affecting their ability to take oral medication (e.g., inability to swallow, chronic diarrhea, intestinal obstruction). Patients could leave the study at the midpoint of the trial period if they withdrew their informed consent. The investigator could exclude the cases that medication violate the treatment protocol and affected the judgment of drug efficacy, and the cases that incomplete data affected the judgment of efficacy and safety.

β-glucan 500 mg was administered orally twice daily combined with the previous protocol (containing PD-1/PD-L1 inhibitor); 1 course was administered every 3 weeks. Treatment was continued until the onset of unacceptable toxicity, disease progression, or death.

Efficacy of the combination therapy was evaluated every 3 cycles according to RECIST 1.1. Complete response (CR) was defined as complete disappearance of the tumor with no new lesions. Partial response (PR) was defined as ≥30% decrease in the longest diameter of the target lesions and no progression in new lesions. Stable disease (SD) was defined as <30% decrease or <20% increase in the target lesions and no progression in new lesions. Progressive disease (PD) was defined as ≥20% increase in the longest diameter of the target lesions or the appearance of new lesions.

The primary study endpoint was the effect of PD-1/PD-L1 inhibitor plus β-glucan on median progression-free survival (mPFS). The secondary endpoint was the effect of this treatment on median overall survival (mOS) and disease control rate.

### Cytokine Assay of Clinic Blood Serum Samples

The cytokines in patients’ blood serum samples were examined with the LEGENDplex Human Essential Immune Response Panel (13-plex) (BD Biosciences), which is a bead-based multiplex assay panel that uses fluorescence-encoded beads suitable for use on various flow cytometers. For this analysis, we collected blood from patients, allowed it to clot for ≥30 min, and centrifuged the blood for 20 min at 1000 × *g* to obtain serum samples. The samples were stored at –20°C. We then added 25 μL of mixed beads, detection antibodies, and SA-PE to each serum sample and standard samples. The samples were shaken at approximately 500 rpm for hours at room temperature. The samples were washed, and 13 key targets were quantified with flow cytometry: IL-1β, IL-2, IL-4, IL-6, IL-10, IL-12p70, IL-17a, TNF-α, TGF-β1, IFN-γ, CXCL10 (IP-10), CCL2 (MCP-1), and CXCL8 (IL-8). Data analysis was then performed using LEGENDplex Data Analysis Software.

### Statistical Analysis

Data are expressed as means and standard errors of the mean (SEM). Comparisons between multiple groups with independent samples were performed using ANOVA, whereas paired t-tests were used to compare patients’ samples before WGP treatment and after WGP treatment. Survival data were plotted as PFS or OS curves. mOS and mPFS values were examined and a pooled effect size and 95% CI were calculated. The thresholds for statistical significance were defined as *p* < 0.05 (*), *p* < 0.01 (**), and *p* < 0.001 (***). Data were analyzed using IBM SPSS statistics (SPSS, 22.0) and GraphPad Prism software (Graphpad, V8.0).

## Results

### Whole Glucan Particle Combined With Anti-PD-1 Therapy Inhibits the Growth of Tumor *In Vivo*

**FIGURE 1:**
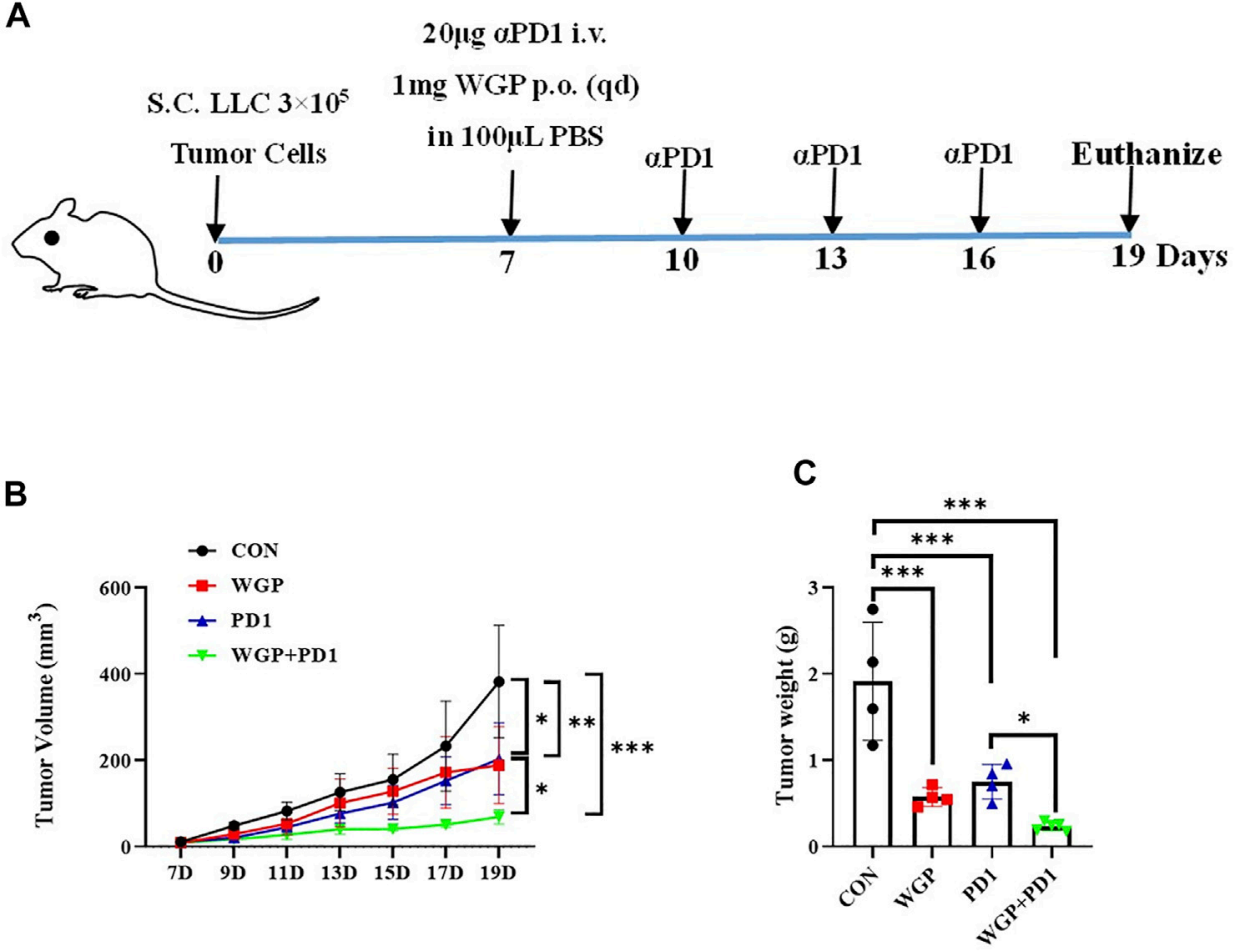
Treatment with WGP and anti-PD1 inhibited the growth of tumor *in vivo*. **(A)** Experimental setup. Mice were treated with 1 mg WGP orally on days 7 to 19; with systemic anti-PD1 antibodies (20 μg) intravenously on days 7, 10, 13, and 16; or with a combination of these treatments. Each group had 4 to 6 mice. **(B)** The tumor masses were measured with a caliper every other day once tumors were palpable, and tumor dimension volumes were calculated. **(C)** The tumor masses were weighed when the mice were euthanized. Treatment groups: control, black; WGP, red; anti-PD1, blue; WGP + anti-PD1, green. *p < 0.05, **p < 0.01, ***p < 0.001.

To investigate the *in vivo* antitumor effects of WGP combined with anti-PD-1 antibody in C57BL/6 mice bearing murine lung LLC, we separated the mice into 4 treatment groups. The WGP and WGP + anti-PD1 groups received 1 mg WGP in 100 μL PBS daily *via* oral administration. The mice in the anti-PD1 and WGP + anti-PD1 groups received 20 μg anti-PD-1 antibody in 100 μL PBS once every 3 days *via* intravenous injection into the tail. The mice in the control group received 100 μL PBS (Figure 1A). We found that tumor-bearing mice treated with combination therapy had a significantly decreased tumor burden when compared with mice treated with single therapy or PBS only. Single therapy also suppressed tumor growth significantly compared with PBS only, but there was no significant difference between WGP only and anti-PD-1 antibody only (Figures 1B,C).

**FIGURE 2:**
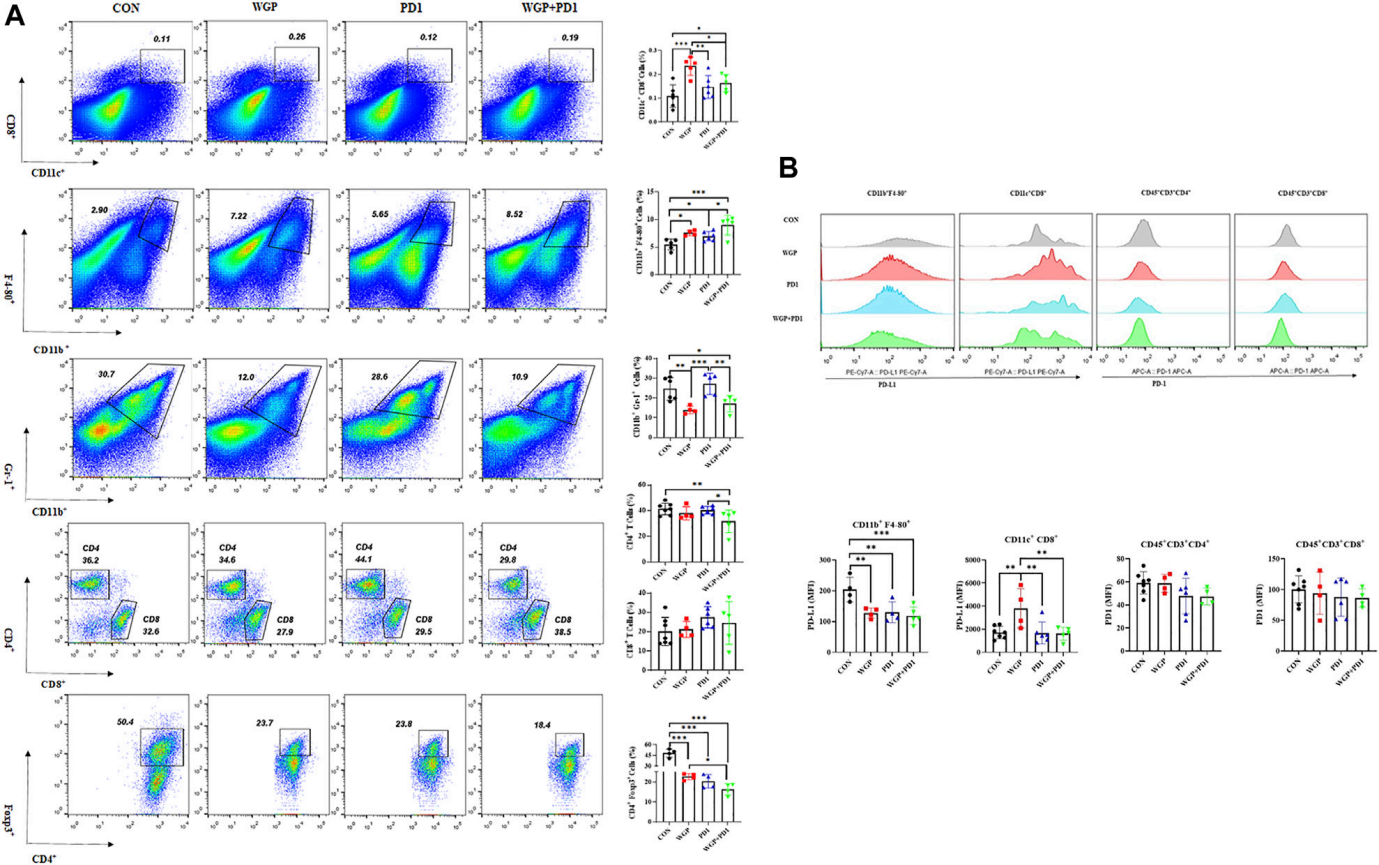
Treatment with WGP and anti-PD1 regulated the frequency of tumor-infiltrating cells. For this analysis, single cell suspensions from tumor samples were stained with fluorochrome-labeled mAbs. **(A)** The concentrations of DCs (CD11c^+^/CD8^+^), macrophages (CD11b^+^/F4-80^+^), MDSCs (CD11b^+^/Gr-1^+^), CD4^+^ T cells/CD8^+^ T cells, and Foxp3^+^/CD4^+^ T cells were determined. **(B)** The expression of PD-L1 and PD1 level on tumor-infiltrating cells were examined. Treatment groups (*n* = 4 to 6 in each group): control, black; WGP, red; anti-PD1, blue; WGP + anti-PD1, green. *p < 0.05, **p < 0.01, ***p < 0.001.

We then examined the tumor-infiltrating cells in the TME and found that with both combination therapy and WGP single therapy, the infiltration of DCs (CD11c^+^/CD8^+^) and macrophages (CD11b^+^/F4-80^+^) was significantly increased, whereas the frequency of MDSCs (CD11b^+^/Gr-1^+^) was significantly decreased (Figure 2A). In addition, combination therapy significantly decreased the proliferation of CD4^+^T cells and Tregs in the tumor (Figure 2A).

**FIGURE 3:**
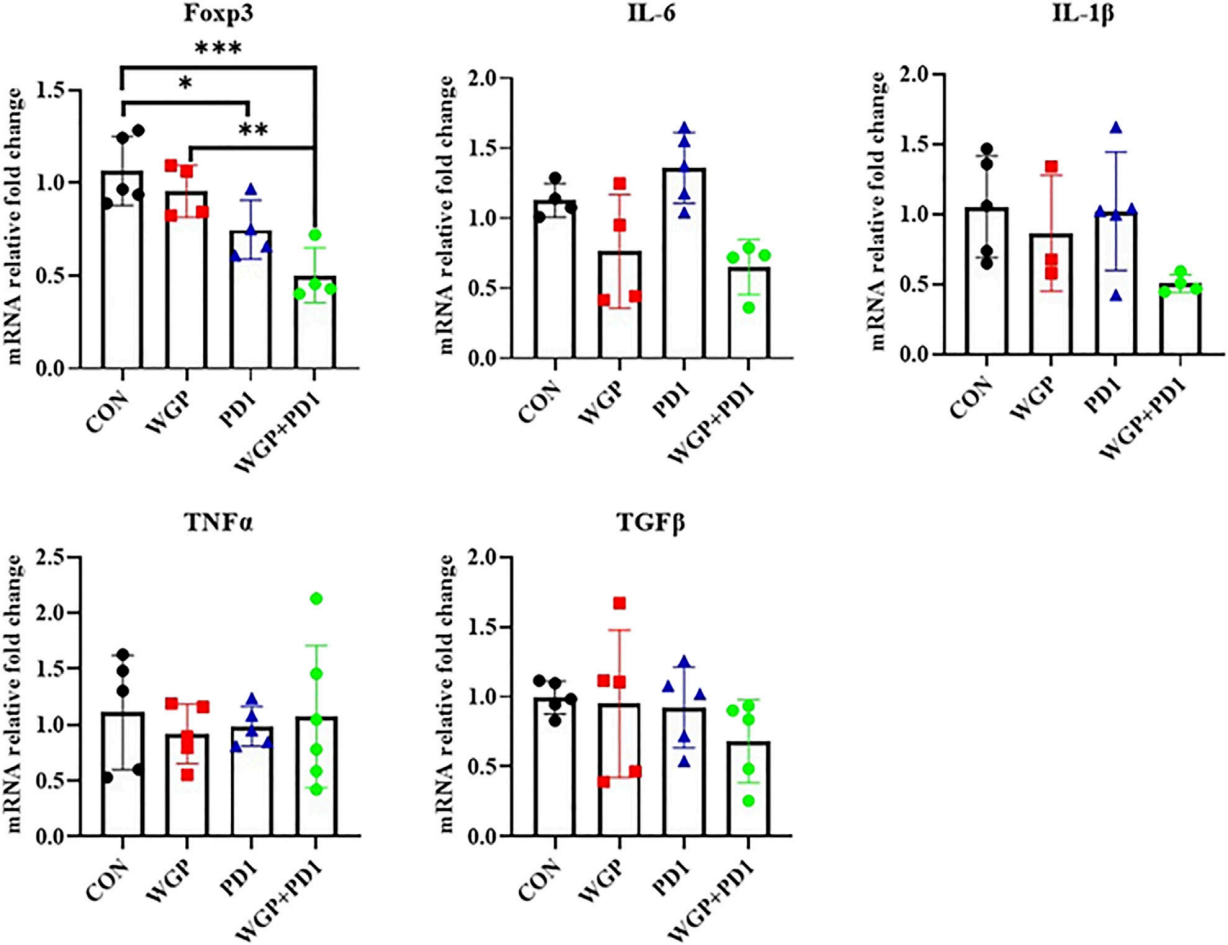
Treatment with WGP and anti-PD1 significantly decreased the mRNA levels of Foxp3 in the tumor microenvironment. Treatment groups (*n* = 4 to 6 in each group): control, black; WGP, red; anti-PD1, blue; WGP + anti-PD1, green. *p < 0.05, **p < 0.01, ***p < 0.001.

To further demonstrate the effects of checkpoint blockade anti-PD-1 antibody therapy *in vivo*, we examined the expression of PD-L1 or PD-1 levels. WGP was found to decrease PD-L1 expression in macrophages but increase PD-L1 expression in DCs. The PD-L1 levels in MDSCs and the PD-1 expression in CD4^+^ and CD8^+^T cells were not significantly altered with single or combination therapy (Figure 2B and Supplementary Figure S1). As shown in Figure 3, the mRNA levels of Foxp3 were significantly decreased in TME after single or combination therapy, with Tregs decreasing in TME (Figure 2A). However, the mRNA levels of IL-6, IL-1β, TNFα, and TGFβ were not significantly changed with or without treatment. These data indicate that WGP treatment expands the influence of anti-PD-1 antibody and is associated with regulating the tumor-infiltrating cells, modulating the suppressive TME, and decreasing Tregs differentiation, thus leading to a delay in tumor progression.

### Whole Glucan Particle Combined With Anti-PD-1/PD-L1 Improves Efficacy in Immune Checkpoint Blockade Resistance Among Patients With Advanced Cancer

The study consisted of 13 patients (9 men, 4 women; mean age, 60 years; range, 47–79 years), all of whom were treated with PD-1/PD-L1 inhibitor plus β-glucan (Supplementary Table S2). Baseline patient characteristics are shown in Table 1.

**TABLE 1:** Baseline characteristics of study patients (N = 13).

Characteristic	No. of patients
ECOG performance status	
0	3
1	9
2	1
Cancer type	
Colon	3
Lung	4
Liver	1
Bladder	1
Breast	1
Pelvic	1
Stomach	1
Kidney	1
Histologic grade	
I-II	2
IV	11
Previous lines of anticancer therapy	
1	2
2	2
3	2
4	3
≥5	4

ECOG, eastern cooperative oncology group.

**FIGURE 4:**
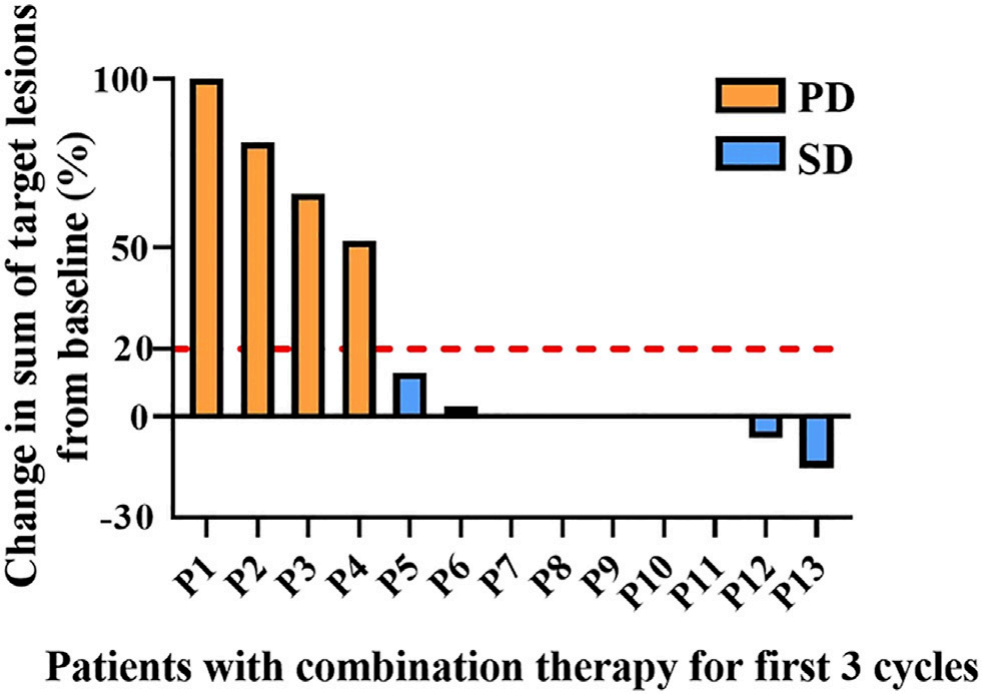
Pooled analysis of disease progression in patients (*n* = 13) treated with WGP and PD-1/PD-L1 inhibitor for the first 3 cycles. Progressive disease (PD), yellow; stable disease (SD), blue.

**FIGURE 5:**
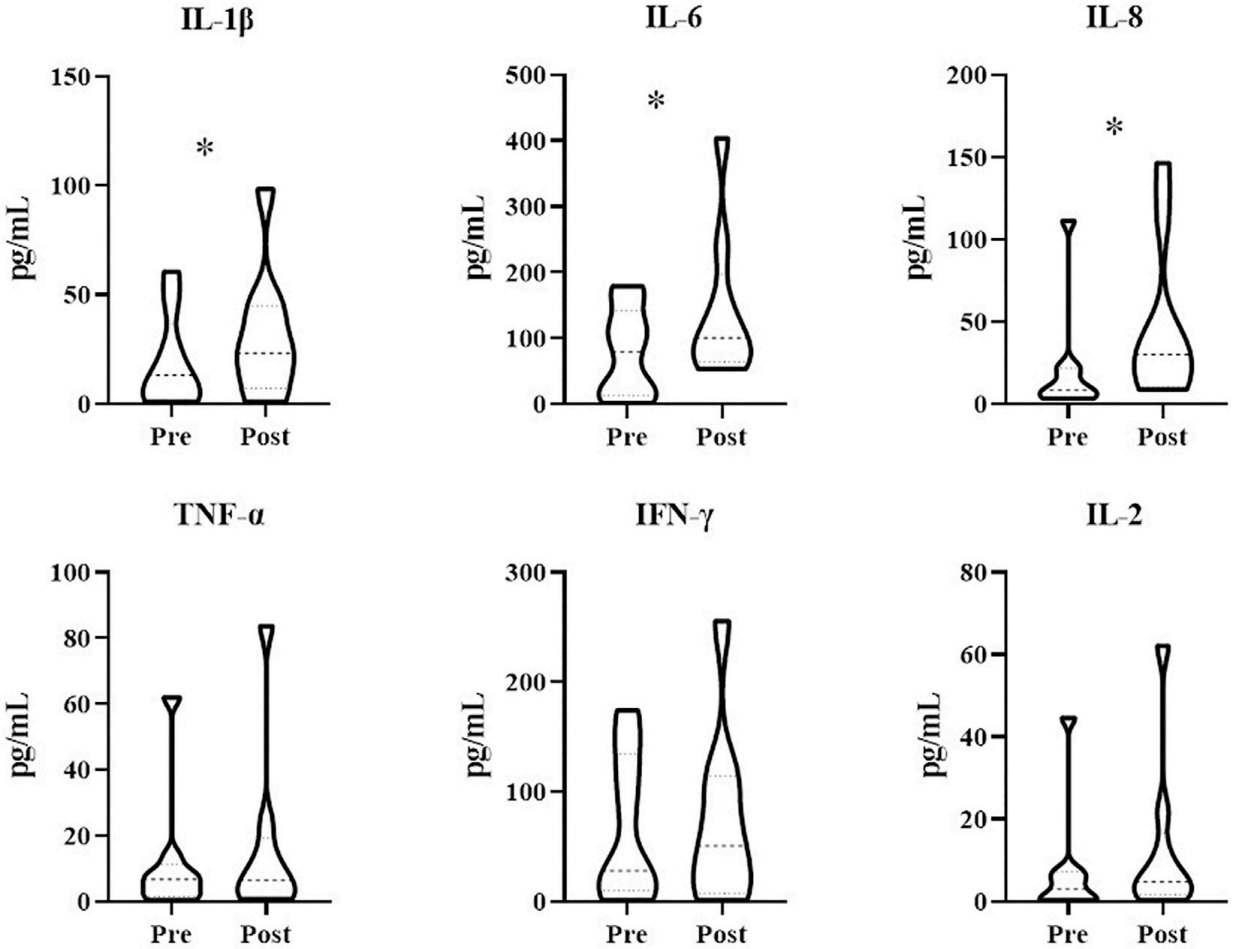
Treatment with WGP increased cytokine levels in patients (*n* = 13) treated with WGP and PD-1/PD-L1 inhibitor. Cytokines in blood serum samples were examined using the LEGENDplex Human Essential Immune Response Panel (13-plex). *p < 0.05.

**FIGURE 6:**
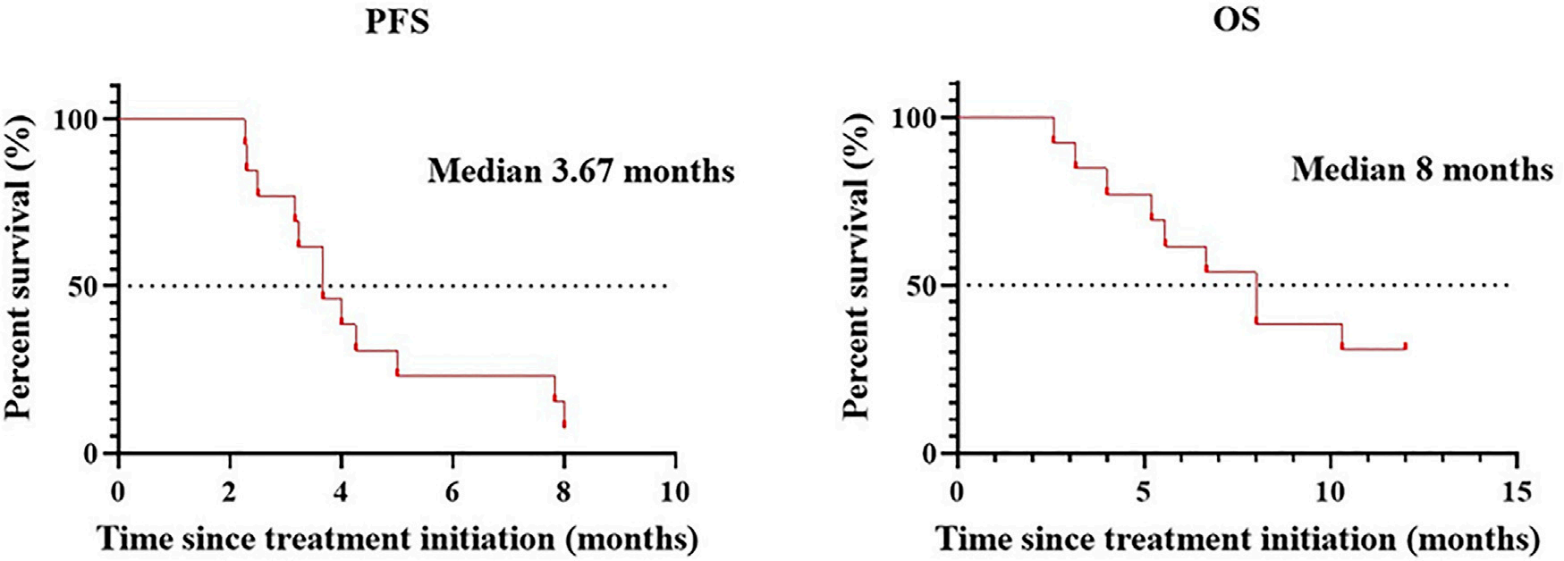
mPFS and mOS in patients (*n* = 13) treated with WGP and PD-1/PD-L1 inhibitor.

Patients treated with combination therapy demonstrated a disease control rate of 69.2%. SD was seen in 9 of the 13 patients (69.2%), and PD was seen in the remaining 4 patients (30.8%). there was no change in the longest diameter of target lesions in 5 of the patients (38.5%), and the tumor size decreased in 2 patients (15.4%) after combination therapy (Figure 4). Cytokine analysis demonstrated that the concentrations of IL-1β, IL-6, and IL-8 were increased in serum samples after combination treatment (Figure 5 and Supplementary Figure S2). The mPFS for the 13 patients was 3.67 months, and the mOS was 8 months (Figure 6). As of 15 December 2021, 4 of the patients were still alive and undergoing continued follow-up.

Immune-related adverse events associated with both the first and rechallenged ICB are shown in Supplementary Table S3. No severe immune-related adverse events occurred in study patients.

## Discussion

The PD-1/PD-L1 pathway plays an essential role in controlling the balance between T-cell activation and tolerance in the TME (Channappanavar et al., 2012), and so PD-1/PD-L1–blocking antibodies are widely used as part of ICB therapy. Nevertheless, PD-1/PD-L1–blocking antibodies have some disadvantages, which we attempted to overcome in this study. In this analysis, we found that WGP β-glucan, a potent immune adjuvant that has been found to improve stimulation of innate and adaptive immune responses (Li et al., 2010), could activate DCs and promote T-cell priming by DCs to augment antitumor immune responses. We found that WGP β-glucan combined with PD-1/PD-L1–blocking antibodies could delay cancer aggression in a lung cancer mouse model and improve mPFS and mOS in patients with advanced cancer who had developed a tolerance to immunotherapy.

Previous research has shown that WGP β-glucan can be phagocytosed by both DCs and macrophages and that oral administration of WGP β-glucan can inhibit tumor development (Ding et al., 2019). In this study, we found that WGP β-glucan combined with PD-1/PD-L1–blocking antibodies inhibited tumor progression more strongly than WGP β-glucan alone or PD-1/PD-L1–blocking antibodies alone. These findings indicate that WGP β-glucan and PD-1/PD-L1–blocking antibodies may use different pathways in their antitumor responses. Furthermore, we found that WGP β-glucan significantly improved the immune response by increasing the concentrations of DCs (CD11c^+^/CD8^+^) and macrophages (CD11b^+^/F4-80^+^) and modulating the suppressive TME by decreasing the concentrations of regulatory T cells and MDSCs. We also observed a significant decrease in Foxp3 mRNA levels in tumor tissues, but no other significant changes were seen for the other molecules studied. Strikingly, both the percentage and mRNA levels of Tregs decreased more than single therapy groups. These data suggest that the pathway involved in WGP β-glucan immune responses is independent of PD-1/PD-L1 blocking.

We observed high levels of PD-L1 expressed on MDSCs and macrophages and high levels of PD-1 expressed on CD4^+^ T cells and CD8^+^ T cells. However, the expression of PD-L1 on macrophages was decreased after combination therapy. Treatment with WGP β-glucan increased the PD-L1 expression on DCs and MDSCs, but this difference was not significant. The blockade of interactions between PD-1 and PD-L1 can enhance response and inhibit tolerance of T cells, playing an essential role in tumor immunity (Hack et al., 2020).

In study patients, we found that IL-1β, IL-6, and IL-8 concentrations were increased after treatment with WGP β-glucan. IL-1β and IL-6, both potent proinflammatory cytokines, are essential in orchestrating the inflammatory response. IL-1β and IL-6 help to coordinate all aspects of local inflammation by attracting and activating cells of the adaptive immune response promptly and transiently, sometimes exacerbating damage in cases of chronic disease and acute tissue injury. IL-1β and IL-6 have a pathological effect on chronic inflammation and autoimmunity (Lopez-Castejon and Brough, 2011). However, the effects of IL-1β and IL-6 on antitumor immune responses are a double-edged sword, as these cytokines can promote tumor development but also abrogate tumor aggression (Castaño et al., 2018). IL-1β and IL-6 are associated with recruitment and differentiation of inflammatory monocytes in the TME (Kaplanov et al., 2019). One recent study found that high IL-1β expression in the primary tumor was associated with improved OS and distant metastasis–free survival among patients with lymph node–positive breast cancer (Weber et al., 2010). As a negative prognostic factor in cancer, IL-6 has been shown to be an important regulator of MDSC accumulation and activation (Tanaka et al., 2014). However, IL-6 is also involved in T-cell priming and differentiation (Weber et al., 2021). IL-8 is a chemokine that plays multiple protumorigenic roles in the TME (Zhang et al., 2020); indeed, many chemokines have functions linked with rapid tumor progression (Morein et al., 2020). However, IL-8 can also increase the expression of inhibitory immune checkpoints such as PD-L1 in immune cells and cancer cells (Wu et al., 2019; Fousek et al., 2021), thus allowing the use of PD-1/PD-L1–blocking antibodies to achieve stronger antitumor effects.

In a recent meta-analysis of 26 studies assessing the efficacy and safety of rechallenge with ICB in patients with solid tumors, the overall PFS was found to be 4.9 months (Inno et al., 2021). In a subgroup of patients who were rechallenged with new ICBs because of PD, the mPFS and mOS were 2.9 and 7.9 months, respectively. In our study, ICB-resistant patients who were treated with β-glucan plus PD-1/PD-L1–blocking antibodies demonstrated a mPFS of 3.67 and a mOS of 8.0 months. These results suggest that β-glucan has promise in reversing resistance to immunotherapy in patients with advanced cancer. Furthermore, no significant toxicities were observed among study patients treated with β-glucan. Furthermore, compared with rechallenge of ICB therapy (Fujita et al., 2018; Niki et al., 2018), add or replace with different classes of ICB (Buchbinder and Desai, 2016; Kaplanov et al., 2019), the combination therapy with WGP β-glucan is efficacy and practicability for patients.

This study has several limitations. First, the patient sample size was small, and the nonrandomized nature of the study may have led to selection bias. In addition, all study patients had advanced cancer and ICB resistance, so most patients were in a poor clinical condition and had suffered from multiple metastases over a long time period. Despite these factors, mPFS and mOS were still found to be improved in patients treated with β-glucan plus PD-1/PD-L1–blocking antibodies.

In summary, we found that the immune adjuvant WGP β-glucan improved the infiltration of both DCs and macrophages in the TME, while PD-1/PD-L1–blocking antibodies promoted the balance of T cells to antitumor effectors. Furthermore, combination therapy with WGP β-glucan plus PD-1/PD-L1–blocking antibodies was found to improve mPFS in ICB-resistant patients with advanced cancer. These results suggest that WGP β-glucan is a potentially useful adjuvant in combination with various ICB therapies in clinical practice.

## Data Availability

The datasets presented in this study can be found in online repositories. The names of the repository/repositories and accession number(s) can be found in the article/Supplementary Material.
