# Supplementary material for: β-Glucan Combined With PD-1/PD-L1 Checkpoint Blockade for Immunotherapy in Patients With Advanced Cancer
Source: Front Pharmacol. 2022 Apr 25;13:887457. doi: 10.3389/fphar.2022.887457 (PMC9084312; doi:10.3389/fphar.2022.887457)
Supplement: Supplementary file 1 [file DataSheet1.docx]

**Supplementary Material**

**Table S1. Primers for Real-Time PCR**

| **Primer** | **Forward** | **Reverse** |
| --- | --- | --- |
| GAPDH | TGTGATGGGTGTGAACCACG | CAGTGAGCTTCCCGTTCACC |
| Foxp3 | AGGAGCCGCAAGCTAAAAGC | TGCCTTCGTGCCCACTGT |
| IL-6 | GAGAAAGAGTTGTGCAATGGC | CCAGTTTGGTAGCATCCATCAT |
| IL-1β | TGCCACCTTTTGACAGTGATG | ATGTGCTGCTGCGAGATTTG |
| TNF-α | TGTAGCCCACGTCGTAGCAAA | CTGGCACCACTAGTTGGTTGT |
| TGF-β | TGCTAATGGTGGACCGCAA | CACTGCTTCCCGAATGTCTGA |

| **Table S2. Patient treatment characteristics** | | | | |  |
| --- | --- | --- | --- | --- | --- |
|  | | | | |  |
| Patient | Sex | | Cancer types | Pre Therapy | Combinational Therapy |
| P1 | Male | Colon cancer | | PD-1(Sintilimab 200mg)  VEGF(Bevacizumab 300mg d1)  Irinotecan(240mg d2) | PD-1（Tislelizumab 200mg d2)  VEGF(Bevacizumab 250mg d1)  β-glucan(500mg bid) |
| P2 | Male | Lung cancer | | PD-L1(Durvalumab 600mg d1/3 weeks)  Anlotinib(12mg qdd1-14) | PD-L1(Durvalumab 600mg d1/3  weeks)  Anlotinib(12mg qdd1-14)  β-glucan(500mg bid) |
| P3 | Female | Pelvic cancer | | PD-1(Sintilimab 200mg)  Gemcitabine(1600mg d1、8) | PD-1(Sintilimab 200mg)  Gemcitabine(1600mg d1、8)  β-glucan(500mg bid) |
| P4 | Male | Lung cancer | | PD-L1(Durvalumab 740mg d1)  VEGF(Bevacizumab 400mg d1)  Albumin paclitaxel(300mg d2) | PD-L1(Durvalumab 740mg d1)  VEGF(Bevacizumab 400mg d1)  Albumin paclitaxel(300mg d2)  β-glucan(500mg bid) |
| P5 | Male | Colon cancer | | PD-L1(Atezolizumab 1200mg)  EGF(Cetuximab 600mg) | PD-L1(Atezolizumab 1200mg)  β-glucan(500mg bid) |
| P6 | Female | Stomach cancer | | PD-1(Sintilimab 200mg)  Oxaliplatin(200mg d1)  Tegafur(40mg bid d1-14) | PD-1（Tislelizumab 200mg d1)  Albumin paclitaxel(200mg d1)  β-glucan(500mg bid) |
| P7 | Male | Bladder cancer | | PD-1(Sintilimab 200mg)  GEMOX | PD-1（Sintilimab 200mg d1)  VEGF(Bevacizumab 400mg d2)  β-glucan(500mg bid) |
| P8 | Female | Lung cancer | | PD-L1(Atezolizumab 1200mg d1)  Paclitaxel(200mg d2) | PD-L1(Atezolizumab 1200mg d1)  Paclitaxel(200mg d2)  β-glucan(500mg bid) |
| P9 | Male | Colon cancer | | PD-1(Toripalimab 240mg d1)  Anlotinib(12mg po d1-14) | PD-1(Toripalimab 240mg d1)  Anlotinib(12mg po d1-14)  β-glucan(500mg bid) |
| P10 | Female | Breast cancer | | PD-L1(Atezolizumab 1200mg)  DS-8201（ENHERTU1200mg) | PD-L1(Atezolizumab 1200mg)  DS-8201（ENHERTU1200mg)  β-glucan(500mg bid) |
| P11 | Male | Live cancer | | PD-1（Camrelizumab 200mg)  Sorafenib(400mg bid) | PD-1（Camrelizumab 200mg)  Sorafenib(400mg bid)  β-glucan(500mg bid) |
| P12 | Male | Kidney cancer | | PD-1（Toripalimab)  Axitinib | PD-1（Tislelizumab 200mg d1)  Apatinib(250mg)  β-glucan(500mg bid) |
| P13 | Male | Lung cancer | | PD-1（Camrelizumab 200mg) | PD-1（Camrelizumab 200mg)  β-glucan(500mg bid)  β-glucan(500mg bid) |

**Table S3. Immune-Related Adverse Events Associated With ICB Treatment**

| Adverse event | First ICB | | Rechallenge ICB | |
| --- | --- | --- | --- | --- |
|  | G1 | G2 ≤ | G1 | G2 ≤ |
| Nausea | 2 | 1 | 1 | 0 |
| Emesis | 1 | 0 | 2 | 0 |
| Diarrhea | 1 | 0 | 0 | 0 |
| Fever | 1 | 0 | 0 | 1 |
| Fatigue | 2 | 1 | 2 | 0 |

ICB, immune checkpoint blockade.

**PD-L1**

**PD1**

**WGP+PD1**

**WGP**

**CON**


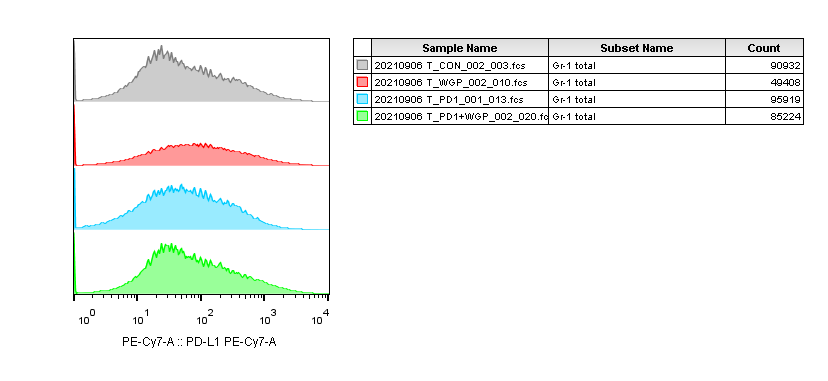


**Figure S1.** The expression of PD-L1 level on MDSCs

**Figure S2.** The cytokine levels in patients (n = 13) pre- or post-treated with WGP and PD-1/PD-L1 inhibitor
